# Surgical procedures and techniques in robot-assisted retrograde para-aortic lymphadenectomy

**DOI:** 10.3389/fonc.2025.1534662

**Published:** 2025-09-09

**Authors:** Xinyou Wang, Ya Li, Jinming Zhu, Jun Wang, Shichao Han, Jing Na

**Affiliations:** ^1^ Obstetrics and Gynecology Department, The Second Affiliated Hospital of Dalian Medical University, Dalian, China; ^2^ Oncology Department, Affiliated Zhongshan Hospital of Dalian University, Dalian, China

**Keywords:** lymphadenectomy, abdominal aorta, membrane bridge, membrane anatomy, robotic surgery

## Abstract

**Background:**

To study the robotic-assisted abdominal aorta lymphadenectomy at the level of the left renal vein, aimed at standardizing and optimizing the surgical procedure.

**Methods:**

All surgical procedures are guided by the theory of membrane anatomy, operating within the intermembrane spaces of embryonic compartments.

**Results:**

Using robotic assistance in a inverted position to perform lymphadenectomy of the abdominal aorta at the level of the left renal vein enables safe and reliable lymph node removal, combined with the concept of membrane anatomy, not only minimizes surgical bleeding but also helps reduce complications, such as vascular and intestinal injuries.

**Conclusion:**

Utilizing robotics to perform lymphadenectomy of the abdominal aorta at the level of the left renal vein can achieve a more meticulous and refined surgical outcome. Precise surgical techniques contribute to standardizing and optimizing surgical procedures, thereby facilitating the learning process.

## 1 Introduction

Lymph node involvement is a critical prognostic factor in gynecologic malignancies, influencing staging, treatment, and outcomes. Consequently, pelvic lymphadenectomy (PLND) and para-aortic lymphadenectomy (PALND), performed for thorough pathological assessment, are fundamental surgical steps in the management of the three major gynecologic cancers: ovarian, endometrial, and cervical carcinoma. In patients with early-stage disease, minimally invasive surgery is the preferred treatment approach, offering equivalent oncologic outcomes to open surgery with less morbidity. Both traditional laparoscopy and robot-assisted laparoscopic surgery are effective for PLND and PALND, with studies generally demonstrating no significant difference in the number of lymph nodes harvested between these approaches.

However, for obese patients, performing PALND via traditional laparoscopy can pose considerable technical challenges. Robot-assisted laparoscopy offers key advantages, including high-resolution, three-dimensional visualization, enhanced instrument dexterity, and greater ergonomic comfort for the surgeon. These features have led to its widespread adoption in both benign and malignant gynecologic surgeries, performed via abdominal and even transvaginal approaches (1). The robotic approach is especially advantageous for high-risk patients, such as those with obesity, as it has been associated with lower complication rates compared to traditional laparoscopy (2).

Due to the limited articulation range of robotic arms, performing true high-level para-aortic lymphadenectomy at the left renal vein level via an antegrade (head-to-foot) approach is challenging (3). Our team has developed a technique involving a 180° rotation of the robotic arms to adopt a retrograde (foot-to-head) operative position. This modification allows the procedure to be performed smoothly, safely, without bleeding, and efficiently, enabling true high-level para-aortic lymphadenectomy at the left renal vein level.

Despite the potential benefits of robotic-assisted surgery, there is a notable lack of detailed, illustrated guides on techniques for high para-aortic lymphadenectomy using robotic platforms. Establishing a standardized approach, especially for high para-aortic dissection, is essential to ensure optimal patient outcomes and procedural reproducibility. Detailed visual and technical documentation would greatly support surgeons in adopting these advanced techniques, allowing them to fully leverage the capabilities of robotic surgery in complex oncologic procedures.

## 2 Indications

Para-aortic lymphadenectomy, performed for staging and debulking purposes, is a critical procedure in the management of gynecologic malignancies. In locally advanced cervical cancer, where positron emission tomography (PET) imaging shows no para-aortic lymph node involvement, pre-treatment staging assessment is crucial. Given the risk of false-negative results with PET scans, this procedure is recommended to accurately evaluate para-aortic lymph node status and to adjust the radiation therapy target fields accordingly. It is also indicated for high-risk endometrial cancer and early-stage ovarian cancer classified as FIGO stage lower than IIB.

## 3 Surgical steps

(Five patients involved in this surgical method so far).

### 3.1 Docking (Tumai)

**Figure 1:**
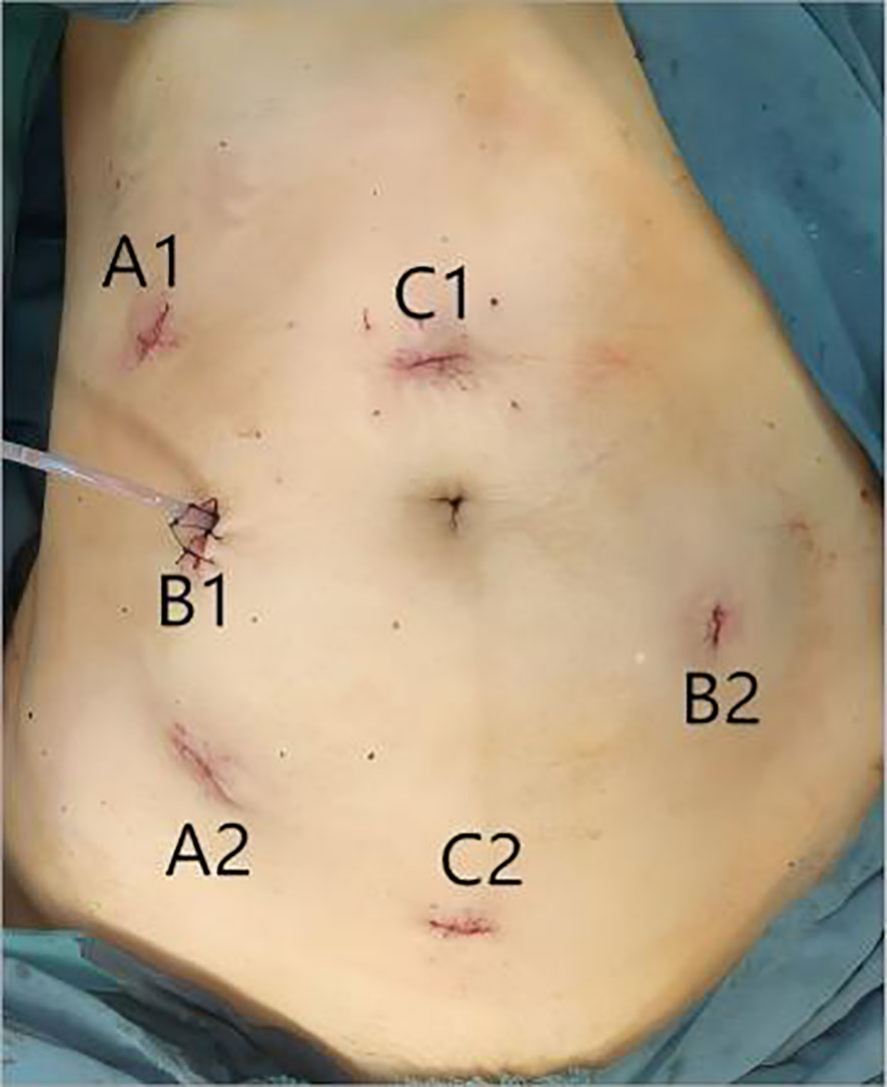
**(A1)** shows the grasping forceps arm puncture point; **(A2)** shows the Assistive puncture point, **(B1)** shows the bipolar arm puncture point; **(B2)** shows the ultrasonic scalpel arm puncture point; **(C1)** shows the Camera arm puncture point in antegrade pelvic operations; **(C2)** shows the Camera arm puncture point.

The robotic arm system is inserted from the tail side and can approach either from the right or the midline. The puncture points are arranged as follows (**Figure 1**):

Camera arm puncture point: Positioned 2–3 cm above the pubic symphysis, avoiding the bladder, with a 12mm trocar inserted.
Bipolar arm puncture point: Corresponding to the ultrasonic scalpel arm’s puncture point in antegrade (head-to-tail) pelvic operations, located approximately 10–13 cm to the right of the camera arm puncture point and angled 15-30° toward the tail side.
Ultrasonic scalpel arm puncture point: Corresponding to the bipolar arm’s puncture point in antegrade pelvic operations, situated approximately 8–10 cm to the left of the camera arm puncture point and angled 15-30° toward the tail side.
Grasping forceps arm puncture point: Corresponding to the assistive puncture point in antegrade pelvic operations, located on the head side along the midline connecting the bipolar arm and camera arm puncture points, at least 5 cm away from the other mechanical arm puncture points.
Assistive puncture point: Corresponding to the grasping arm’s puncture point in antegrade pelvic operations, located 6–8 cm to the right of the ultrasonic scalpel arm puncture point and angled 15-30° toward the tail side.

### 3.2 Exposure of the para-aortic area

Using an ultrasonic scalpel, sharply dissect the connection between the ascending colon mesentery and the retroperitoneum, referred to as the “membrane bridge (4–6).” Proceed with sharp dissection to separate the ascending colon mesentery and the duodenum from the para-aortic area, retracting them laterally to expose the para-aortic region.

The assistive arm retracts the intestinal cranially and ventrally. Using the ultrasonic scalpel, sharply dissect the retroperitoneum under the duodenum in a cranial direction. After transecting the ligament of Treitz, the left renal vein is exposed.

**Figure 2:**
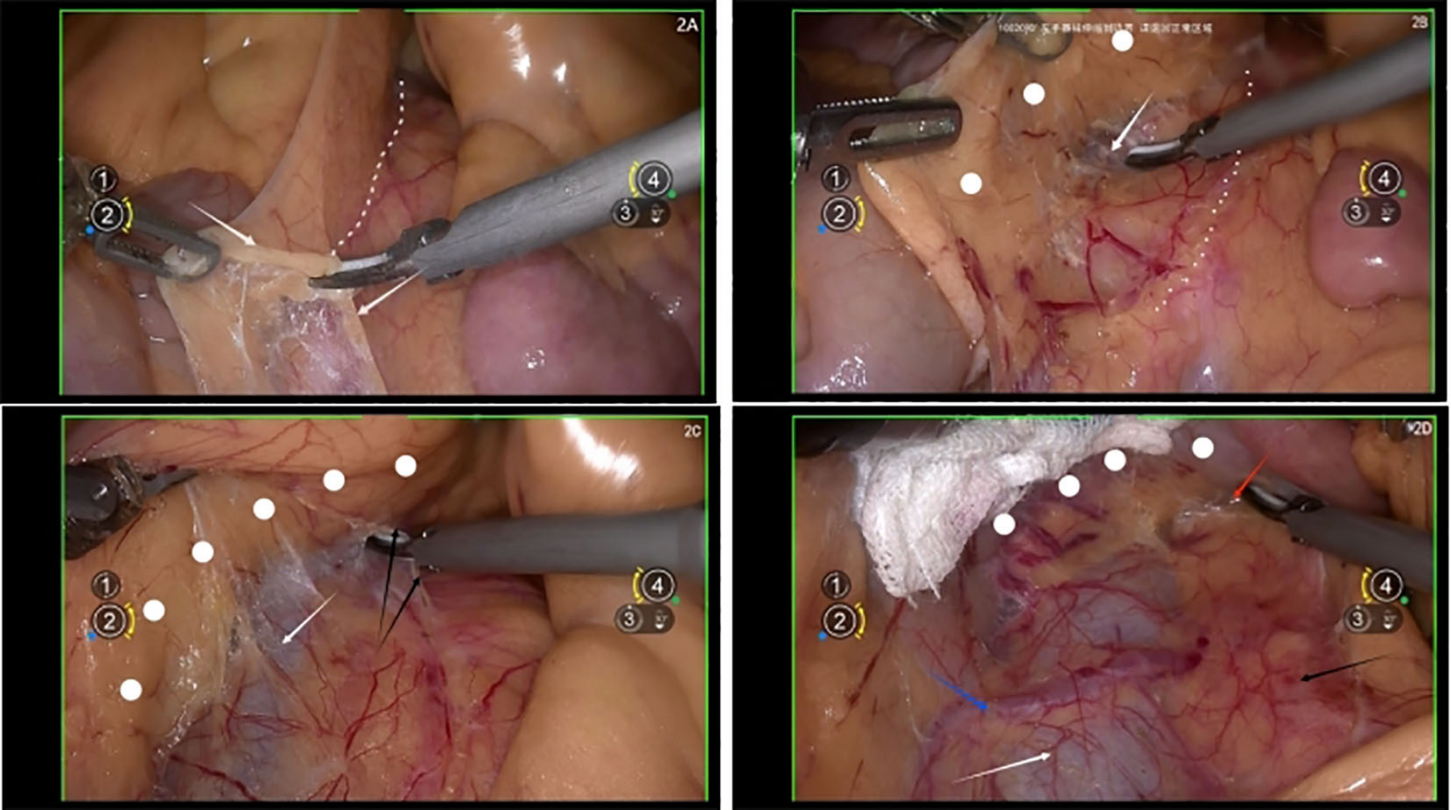
**(A)** white arrow shows the retroperitoneum, white dotted line shows the connection between the ascending colon mesentery and the retroperitoneum, referred to as the “membrane bridge”; **(B)** white arrow shows the inferior vena cava, white dotted line shows the retroperitoneum, white dots show the ascending colon mesentery; **(C)** white arrow shows the inferior vena cava, black arrows show the Treitz ligament, white dots show the ascending colon mesentery; **(D)** white arrow shows the inferior vena cava, white dots show the ascending colon mesentery, black arrow shows the abdominal aorta, red arrow shows the left renal vein, blue arrow shows the right ovary artery.

Through surgical practice, our team has observed that the left renal vein is typically located at the level of the ligament of Treitz or at the junction of the retroperitoneum and the inferior mesenteric vein (**Figure 2**).

During duodenal exposure, it is crucial to use laparoscopic gauze to isolate and protect the duodenum while elevating it with the assistive arm to avoid injury.

### 3.3 Suspending the retroperitoneum and mesentery of the ascending and descending colon

**Figure 3:**
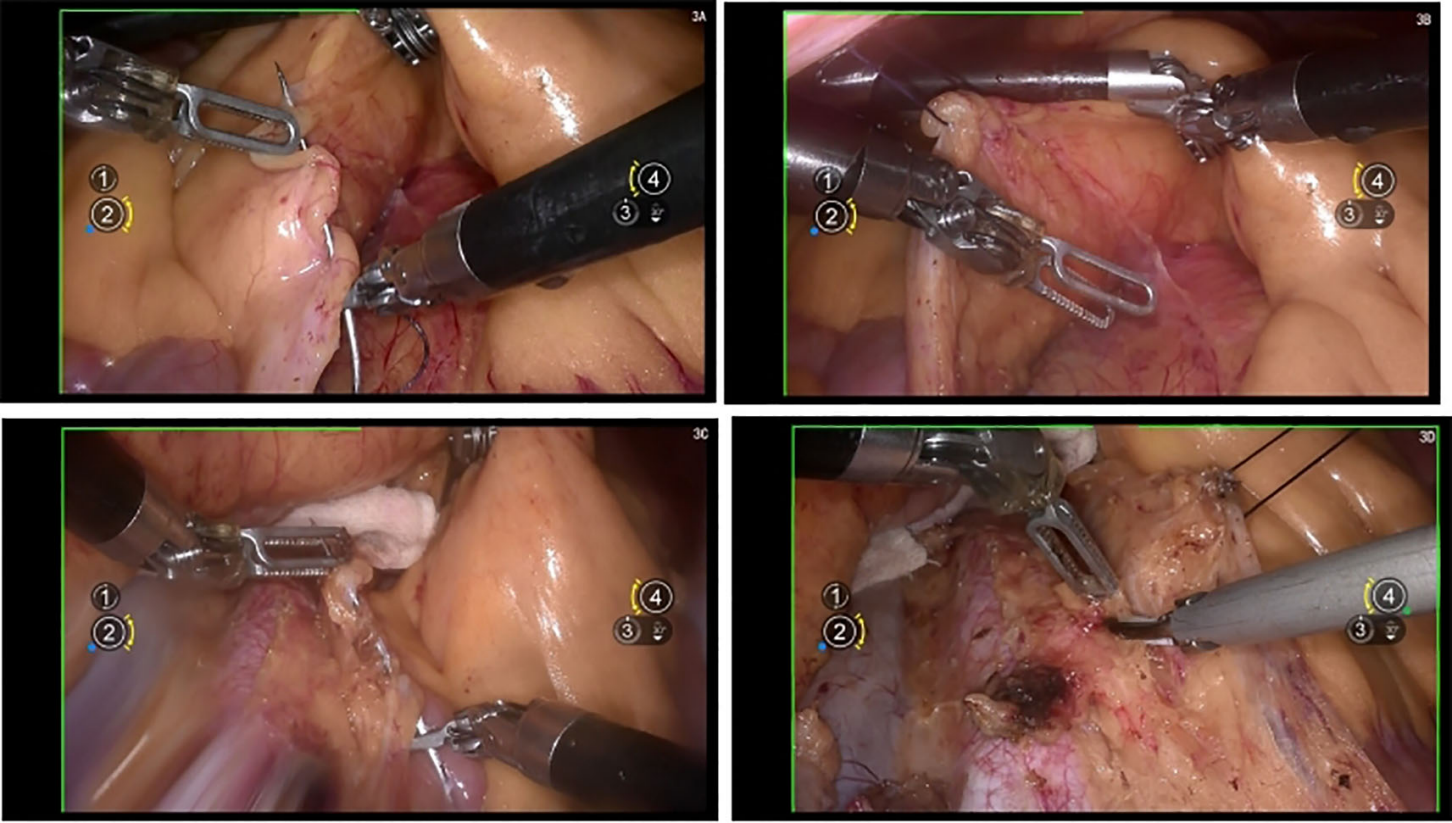
**(A, B)** show the suspending the retroperitoneum and mesentery of the ascending colon; **(C, D)** shows the suspending the retroperitoneum and mesentery of the descending colon.

The mesentery and the incised posterior peritoneum were sutured and suspended to the abdominal wall using absorbable materials, thereby achieving optimal exposure of the surgical field (**Figure 3**).

### 3.4 To enhance surgical field exposure, the mobilized ascending and descending colons can be suspended and retracted

A 1–0 Vicryl suture is used to secure the peritoneum and mesentery, which is then passed through the abdominal wall. This technique facilitates the lateral and ventral retraction of the mesentery and colon, improving visualization of the surgical field.

### 3.5 Resection of lymph nodes on the surface of the inferior vena cava and between the inferior vena cava and the abdominal aorta

#### 3.5.1 Key considerations during this procedure

##### 3.5.1.1 Bridging vessels

**Figure 4:**
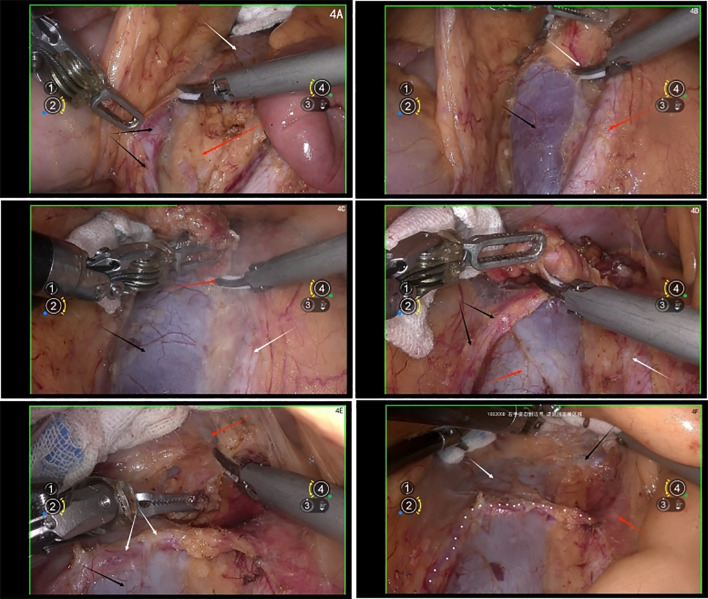
**(A)** white arrow shows the inferior vena cava (IVC), black arrows show the ureter, red arrow shows the lymph nodes on the right common iliac vein; **(B)** white arrow shows the bridge vessel between the IVC and the lymph nodes, black arrow shows the inferior vena cava, red arrow shows the right common iliac artery; **(C)** black arrows show the IVC, white arrow shows the abdominal aorta, red arrow shows the resection of the lymph nodes between IVC and abdominal aorta; **(D)** black arrows show the right ovary artery, red arrow shows the IVC, white arrow shows the abdominal aorta; **(E)** red arrow shows the left renal vein, white arrows show the right ovary artery, black arrow shows the IVC; **(F)** black arrow shows the left renal vein, red arrow shows the abdominal aorta, white arrow shows the IVC, white dotted line shows right ovary artery.

Lymph nodes are often associated with bridging vessels between the IVC and the lymph nodes. These vessels should be adequately sealed with bipolar coagulation before division or secured with titanium clips prior to transection; Crossing arteries: be cautious with artery crossing the surface of the IVC, it could be the right ovarian artery or the right adrenal artery. Thoroughly review preoperative imaging to confirm the organ supplied by the artery before proceeding with appropriate management; Ureter identification: Before lymph node resection, always identify the right ureter and retract it laterally to avoid injury (**Figure 4**).

### 3.6 Resection of lymphatic fatty tissue on the surface of the abdominal aorta

**Figure 5:**
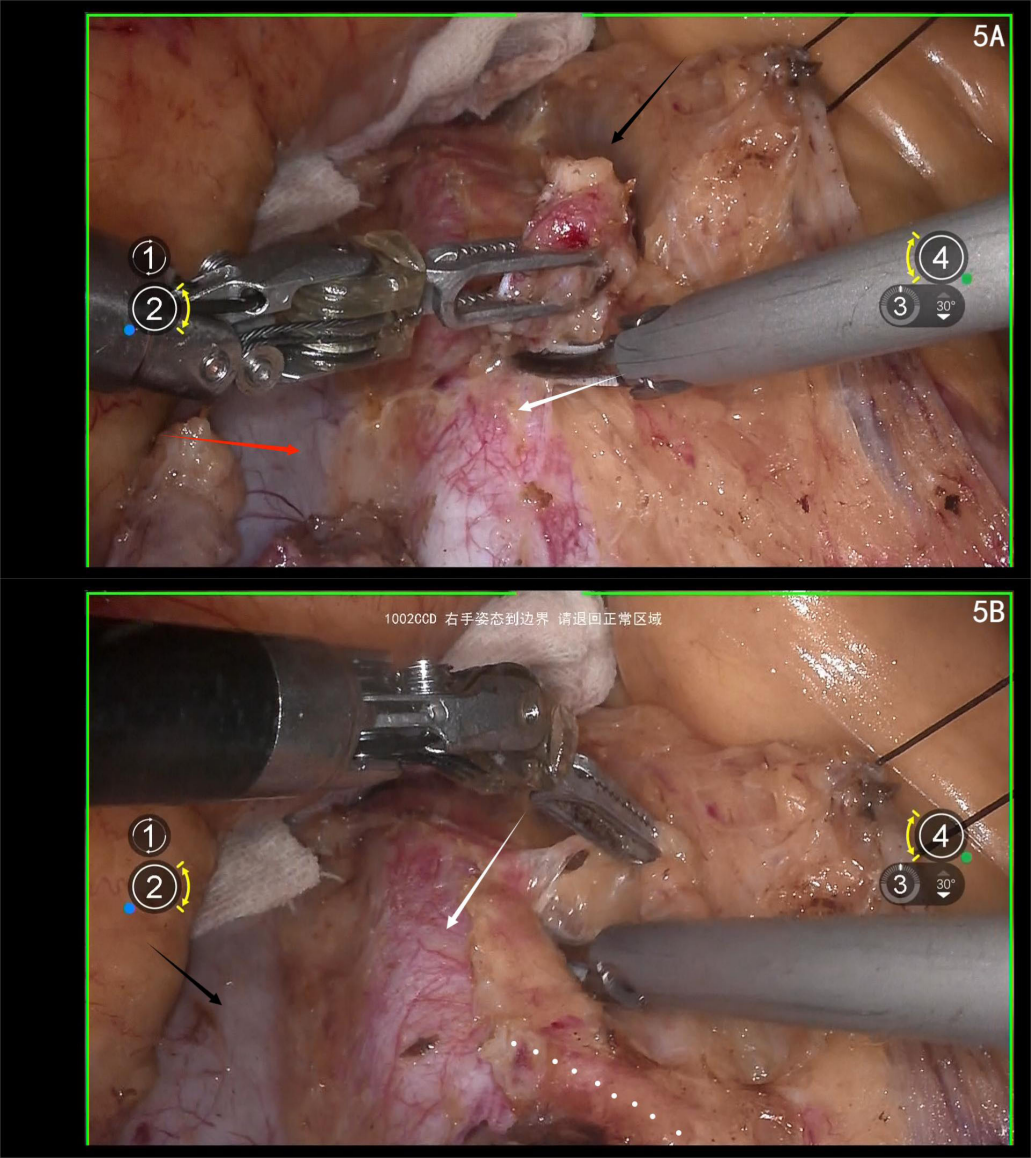
**(A)** white arrow shows the abdominal aorta; red arrow shows the IVC; black arrow shows the inferior mesenteric vein(IMV); **(B)** white arrow shows the abdominal aorta, black arrow shows the IVC, white dotted line shows the IMA.

Using ultrasonic scalpel, sharply dissect the lymphatic fatty tissue on the surface of the abdominal aorta from the caudal to the cranial direction. Take extra care when approaching the root of the inferior mesenteric artery (IMA). Ensure the procedure is performed under direct vision to prevent injury to the IMA (**Figure 5**).

### 3.7 Resection of lymphatic fatty tissue in the cranial region of the IMA

Exposure of the lateral boundary of the lymphatic fatty tissue in the cranial region of the IMA: Using an ultrasonic scalpel dissection in the intermembrane space between the hindgut embryonic compartment and the lymphatic fatty tissue, expanding dorsally and longitudinally. This process gradually exposes the left ovarian vein and the renal fat tissue of the metanephric compartment. The cranial ureter typically enters the perirenal fat tissue and does not require explicit exposure.

**Figure 6:**
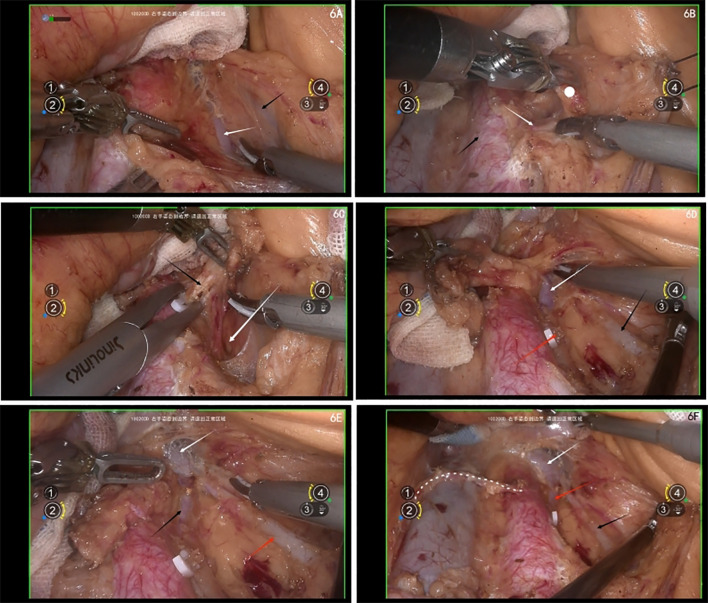
**(A)** white arrow shows the left ovary vein, black arrow shows the inferior mesenteric vein(IMV); **(B)** white arrow shows IMA, black arrow shows the abdominal aorta, white dot shows the lymph nodes; **(C)** white arrow shows the intermembrane space between hindgut embryonic compartment and the lymphatic fatty tissue, black arrow shows the left ovary artery; **(D)** white arrow shows the left ascending lumbar vein; black arrow shows the left ovary vein, red arrow shows the cut end of the left ovary artery; **(E)** white arrow shows left renal vein, black arrow shows the left ascending lumbar vein, red arrow shows the left ovary vein; **(F)** white arrow shows left renal vein, black arrow shows the left ovary vein, red arrow shows the left ascending lumbar vein, white dashed line shows the right ovary artery.

Sharp dissection close to the abdominal aorta should be performed with the ultrasonic scalpel. If the left ovarian artery is encountered, secure it with vascular clips before transection (ensure preoperative imaging has ruled out the possibility of it being the left adrenal artery) (**Figure 6**).

#### 3.7.1 Key points for resection

As dissection nears the inferior border of the left renal vein, careful separation is essential due to the frequent presence of lumbar veins or ascending lumbar veins, to avoid the injury of these veins. Injuries to these veins can result in significant bleeding, which may be difficult to control. Hemostasis typically involves the use of vascular clips.

### 3.8 Excision of lymphatic fat tissue in the caudal region of the IMA

Key points for removing lymphatic fat tissue in this region include:

Retract the ureter of the metanephric compartment outward, away from the lymphatic fat tissue; Take care to avoid injuring the paired lumbar arteries and veins, which are typically located near the level of the IMA in this region; Avoid to injury the lumbar sympathetic trunk and sympathetic ganglia; When mobilizing the IMA, do not approach its root too closely, preserve the mesentery near the root as much as possible, and avoid excessive lateral traction on the IMA to prevent avulsion or rupture of its root.

## 4 Discussion

**Figure 7:**
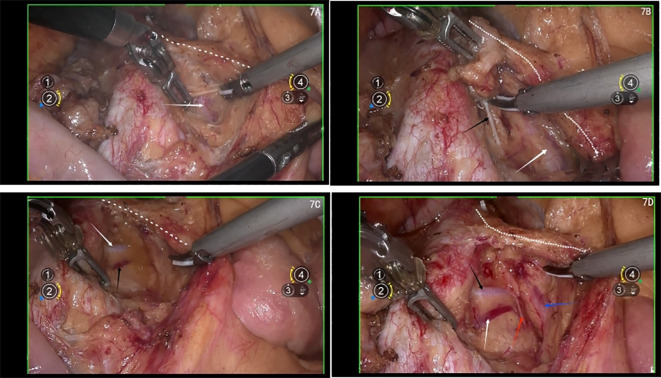
**(A)** white arrow shows the left ureter, white dashed line shows the IMA; **(B)** white arrow shows the left ureter, white dashed line shows the IMA, black arrow shows the right lumbar sympathetic nerve, **(C)** white arrow shows the right lumbar artery, black arrow shows the right lumbar vein, white dashed line shows the IMA; **(D)** white arrow shows the right lumbar vein; black arrow shows the right lumbar artery, red arrow shows the right lumbar sympathetic nerve, blue arrow shows the left ureter.

Lymphadenectomy is an indispensable component of gynecological oncology surgery (7–9). However, performing lymphadenectomy in the para-aortic region at the level of the left renal vein is particularly challenging due to its complex anatomy and limited exposure. The widespread adoption of robotic-assisted technology has significantly reduced the difficulty of operating in this region (10–13). In robotic-assisted surgery, the robotic arms are positioned on both sides of the surgical field and are independently controlled by the surgeon’s left and right hands, offering an experience similar to open surgery, which makes the technique easier to learn (**Figure 7**). Additionally, robotic-assisted laparoscopy provides three-dimensional magnified visualization, flexible wrist-like movements of the robotic arms, tremor elimination, and motion scaling, enabling surgeons to perform complex surgical maneuvers with greater precision in a minimally invasive setting. Moreover, robotic-assisted surgery ensures stable control and reduces the risk of chronic musculoskeletal injuries in surgeons caused by prolonged procedures in conventional laparoscopy, particularly during lengthy surgeries.

Some scholars have adopted an extraperitoneal approach for para-aortic lymphadenectomy, achieving favorable results (14–16). However, regardless of whether the left or right extraperitoneal approach is used, it remains challenging to completely remove lymph nodes on the contralateral side. A major complication following lymphadenectomy is the development of lymphoceles. In the extraperitoneal approach, the peritoneum remains intact, preventing lymphatic fluid from draining into the pelvic or abdominal cavities. This increases the likelihood of retroperitoneal lymphoceles and, in some cases, even lymphatic abscesses.

The advantages of robotic surgery are similar to those commonly reported in other procedures, particularly the improvement in surgeon comfort and assistance. The robot allows the operator to independently control the camera. Additionally, the 3D, 10-15x magnified surgical field enhances precision during surgical maneuvers. The stability and tremor-free operation of the robotic arms ensure consistent and steady performance throughout the procedure (16).

The suspension of the colonic mesentery combined with retrograde high para-aortic lymphadenectomy provides direct visualization of critical anatomical structures, such as the inferior vena cava, abdominal aorta, and left renal vein, meeting the requirements for the surgical field. This approach allows for the thorough removal of lymph nodes from the surface of the inferior vena cava, the space between the inferior vena cava and the abdominal aorta, and the left side of the abdominal aorta. It addresses the limitations of the extraperitoneal approach, which primarily enables effective lymphadenectomy only on the same side as the surgical approach. Moreover, in extraperitoneal para-aortic lymphadenectomy, inadequate exposure of the contralateral inferior vena cava or abdominal aorta can make vascular repair extremely challenging in the event of contralateral vascular injury.

## 5 Conclusion

Performing retrograde high para-aortic lymphadenectomy using a robotic system is a well-established and highly adaptable technique. By offering a detailed explanation of the surgical steps, this method can be quickly mastered by a broad range of surgeons, allowing more patients to benefit from its implementation.

## Data Availability

The original contributions presented in the study are included in the article/supplementary material. Further inquiries can be directed to the corresponding authors.
